# Overexpression of *Fgfr2c* causes craniofacial bone hypoplasia and ameliorates craniosynostosis in the Crouzon mouse

**DOI:** 10.1242/dmm.035311

**Published:** 2018-11-09

**Authors:** Kevin K. L. Lee, Emma Peskett, Charlotte M. Quinn, Rosanna Aiello, Liliya Adeeva, Dale A. Moulding, Philip Stanier, Erwin Pauws

**Affiliations:** 1 Developmental Biology and Cancer Programme, UCL Great Ormond Street Institute of Child Health, 30 Guilford Street, London WC1N 1EH, UK; 2 Genetics and Genomic Medicine Programme, UCL Great Ormond Street Institute of Child Health, 30 Guilford Street, London WC1N 1EH, UK; 3 ICH GOSH Light Microscopy Core Facility, UCL Great Ormond Street Institute of Child Health, 30 Guilford Street, London WC1N 1EH, UK

**Keywords:** FGFR2, FGF, Craniosynostosis, Cleft palate, Crouzon, ERK

## Abstract

FGFR2c regulates many aspects of craniofacial and skeletal development. Mutations in the *FGFR2* gene are causative of multiple forms of syndromic craniosynostosis, including Crouzon syndrome. Paradoxically, mouse studies have shown that the activation (*Fgfr2c*^C342Y^; a mouse model for human Crouzon syndrome), as well as the removal (*Fgfr2c*^null^), of the FGFR2c isoform can drive suture abolishment. This study aims to address the downstream effects of pathogenic FGFR2c signalling by studying the effects of *Fgfr2c* overexpression. Conditional overexpression of *Fgfr2c* (*R26R*^Fgfr2c;βact^) results in craniofacial hypoplasia as well as microtia and cleft palate. Contrary to *Fgfr2c*^null^ and *Fgfr2c*^C342Y^, *Fgfr2c* overexpression is insufficient to drive onset of craniosynostosis. Examination of the MAPK/ERK pathway in the embryonic sutures of *Fgfr2c*^C342Y^ and *R26R*^Fgfr2c;βact^ mice reveals that both mutants have increased pERK expression. The contrasting phenotypes between *Fgfr2c*^C342Y^ and *R26R*^Fgfr2c;βact^ mice prompted us to assess the impact of the *Fgfr2c* overexpression allele on the Crouzon mouse (*Fgfr2c*^C342Y^), in particular its effects on the coronal suture. Our results demonstrate that *Fgfr2c* overexpression is sufficient to partially rescue craniosynostosis through increased proliferation and reduced osteogenic activity in E18.5 *Fgfr2c*^C342Y^ embryos. This study demonstrates the intricate balance of FGF signalling required for correct calvarial bone and suture morphogenesis, and that increasing the expression of the wild-type FGFR2c isoform could be a way to prevent or delay craniosynostosis progression.

## Abstract (precis)

**Summary:** Increased levels of FGFR2c cause craniofacial bone hypoplasia, microtia and cleft palate, but not craniosynostosis. Introduction of an extra *Fgfr2c* allele into a mouse model for Crouzon syndrome partially rescues the craniosynostosis phenotype.

## INTRODUCTION

Normal craniofacial development is a precisely coordinated process that involves the modelling of a framework supporting the soft tissues of the head, in particular the brain. During normal development, growth of the brain is possible because suture tissue separates the calvarial bones and allows for skull vault expansion. Craniosynostosis, a common birth defect with an incidence of 1:2500, is characterised by the loss of suture tissue followed by premature fusion of calvarial bones. This results in the restriction of brain growth and is often associated with dramatic dysmorphology of the skull and face. A significant number of craniosynostosis cases are syndromic and associated with additional skeletal phenotypic features. Many of these syndromes are caused by mutations in FGF pathway genes (Johnson and Wilkie, 2011).

FGF signalling is important for cellular proliferation, differentiation and survival. Receptor activation allows signals to be conveyed through the RAS-MAPK, P13K-AKT and PLCγ-PKC cascades via a series of protein intermediates (Ornitz and Itoh, 2015; Eswarakumar et al., 2005). FGF receptors (FGFRs) are highly conserved receptor tyrosine kinases (RTKs) located on the cell membrane with intracellular and extracellular domains. Tissue specific isoforms are produced by alternative splicing, affecting the third extracellular immunoglobulin-like loop (DIII) of FGFR1-3. The FGFR ‘IIIb’ and ‘IIIc’ isoforms differ only in the C-terminal half of DIII, which is encoded by exons 8 or 9 for the IIIb and IIIc splice forms, respectively (Eswarakumar et al., 2005; Ornitz and Itoh, 2015). Furthermore, the expression of the isoforms are tissue specific and critical for establishing paracrine reciprocal signalling loops: while the IIIb isoform is commonly expressed in epithelial cells and receives FGF ligand from the mesenchyme, the IIIc is expressed in mesenchymal cells and receives FGF ligand from the neighbouring epithelium (Orr-Urtreger et al., 1993).

Mutations affecting the *FGFR2* gene can cause a spectrum of craniofacial phenotypes commonly associated with growth dysplasia, mid-facial hypoplasia, coronal synostosis, orbit dysmorphology and cleft palate (Wilkie, 1997). Notably, synostosis of the coronal suture is a hallmark of human Crouzon and Pfeiffer syndromes (Johnson and Wilkie, 2011). Crouzon syndrome is most commonly caused by a point mutation in exon 9 of the *FGFR2* gene, is autosomal dominantly inherited and exclusively affects the IIIc isoform (Reardon et al., 1994). The substitution of a cysteine to a tyrosine residue (p.C342Y) results in the stabilisation of intermolecular disulphide bonds in the receptor extracellular domains that lead towards ligand independent receptor activation, and is often referred to as a gain-of-function (GOF) mutation (Reardon et al., 1994).

In the developing mouse cranial vault, *Fgfr2* is expressed in the osteogenic fronts of the calvarial bones, but the specific cellular localisation of the different FGFR2 isoforms remains elusive due to their high sequence homology (Johnson et al., 2000; Iseki et al., 1997). FGFR2c function is commonly associated with craniofacial and skeletal development, as genetic mutation in mouse models leads to a series of craniofacial malformations and additional skeletal dysmorphology (Lee et al., in press). Deletion of the FGFR2c isoform (*Fgfr2c*^null^) results in skeletal hypoplasia and craniosynostosis owing to imbalances in osteoprogenitor proliferation and differentiation in the endochondral and intramembranous skeleton (Eswarakumar et al., 2002). The most common coding mutation responsible for human Crouzon syndrome has been introduced into the mouse (*Fgfr2c*^C342Y^) to study the pathogenesis of the disease in more detail (Eswarakumar et al., 2004). The craniofacial phenotype in these mice includes brachycephaly due to postnatal coronal craniosynostosis, as well as a short snout caused by mid-facial hypoplasia (Eswarakumar et al., 2004). Contrary to *Fgfr2c*^null^ mice, increased numbers of osteoprogenitor cells were observed, but, paradoxically, synostosis of the coronal suture was also present (Eswarakumar et al., 2004). Recently, it was shown that increased levels of MAPK/ERK signalling downstream of FGFR2c are present in suture osteoprogenitors in *Fgfr2c*^C342Y^, but not *Fgfr2c*^null^, mice (Pfaff et al., 2016). In addition, hemizygous mutants (*Fgfr2c*^C342Y/−^) show a more severe craniofacial phenotype, demonstrating that lowering the *Fgfr2c* expression levels does not alleviate the features associated with FGFR2c activation (Pfaff et al., 2016). Altogether, the mechanism behind pathogenic FGFR2c signalling in the coronal suture is complex, as indicated by the observation that coronal synostosis is a notable phenotype to both activation (*Fgfr2c*^C342Y^) and loss (*Fgfr2c*^null^) of FGFR2 signalling (Eswarakumar et al., 2002, 2004). Furthermore, analysis of the cleft palate phenotype in *Fgfr2c*^C342Y^ mice demonstrates that activation, as well as inhibition, of FGF receptor signalling causes delayed palatal shelf growth and elevation (Snyder-Warwick et al., 2010). These results imply that the downstream interpretation of FGFR2c signalling can be different from the activity of the receptor itself, and the phenotype elicited might not be a direct translation of an overactive FGFR2c signalling pathway.

The complexities of FGFR2c signalling prompted us to delineate the molecular basis for signalling misregulation further by studying *Fgfr2c* receptor overexpression using a conditional allele that allows tissue-specific induction (i.e. *R26R*^Fgfr2c-flox^). This study reports that the phenotypic consequences of *Fgfr2c* overexpression show similarities as well as differences to those found in *Fgfr2c*^C342Y^ and *Fgfr2c*^null^ mice. Here, we interrogated the biochemical and transcriptional FGFR2 pathway activity in the coronal sutures prior to the onset of synostosis, and show that both constitutive activation and overexpression of FGFR2c results in increased levels of ERK phosphorylation *in vivo*. Furthermore, we provide evidence that increasing the expression of a wild-type (WT) *Fgfr2c* allele can ameliorate the craniosynostosis phenotype in *Fgfr2c*^C342Y^ Crouzon mice.

## RESULTS

### *Fgfr2c* overexpression causes growth restriction, microtia and cleft palate

**Fig. 1.**
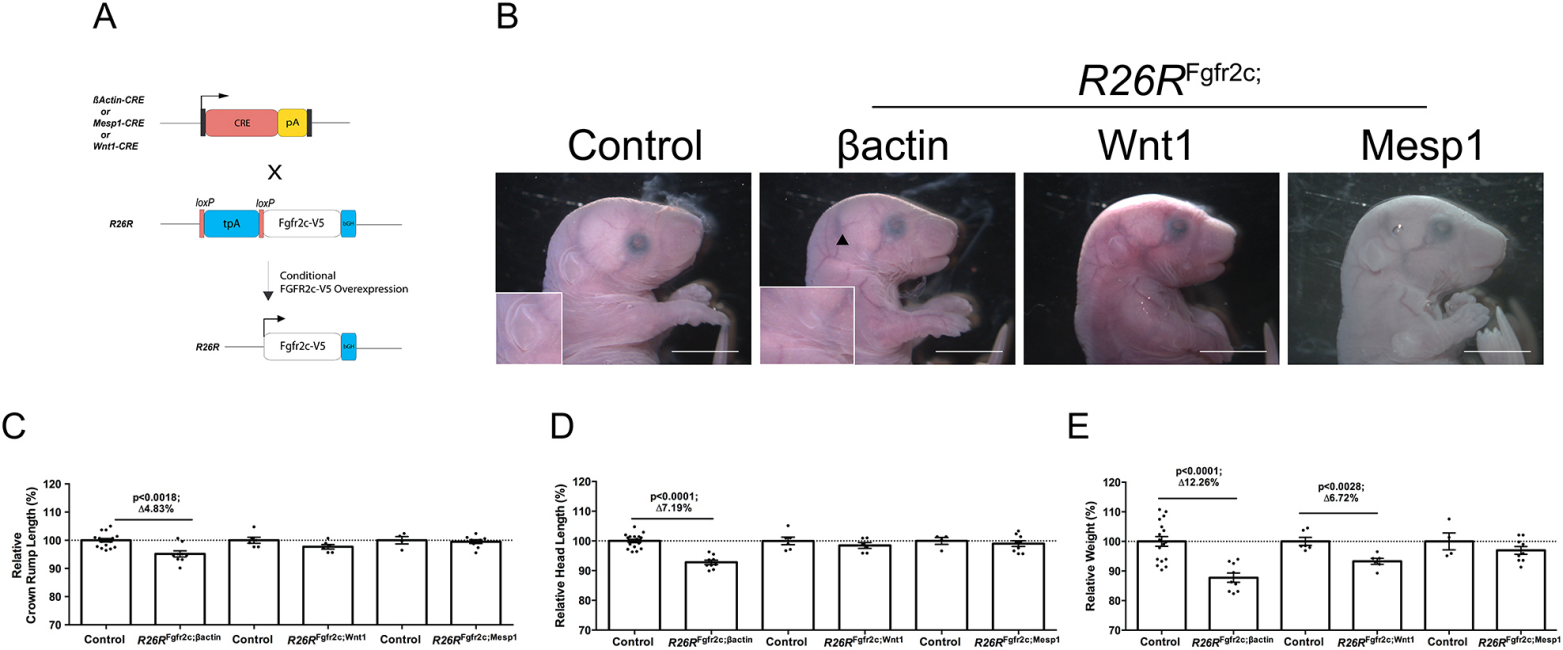
**Gross phenotype of *Fgfr2c* overexpression in E18.5 mutants.** (A) Breeding strategy for conditional *Fgfr2c* overexpression. (B) Microtia (arrowhead+inset) was observed in *R26R*^Fgfr2c;βact^ only. (C) Skeletal hypoplasia in *R26R*^Fgfr2c;βact^ (*P*<0.0018). (D) Craniofacial hypoplasia in *R26R*^Fgfr2c;βact^ (*P*<0.0001). (E) *R26R*^Fgfr2c;βact^ and *R26R*^Fgfr2c;Wnt1^ display significant reductions in weight (*P*<0.0001 and *P*<0.0028, respectively). Statistics: Student’s *t*-test with Welch's correction. Scale bars: 5 mm.

**Fig. 2.**
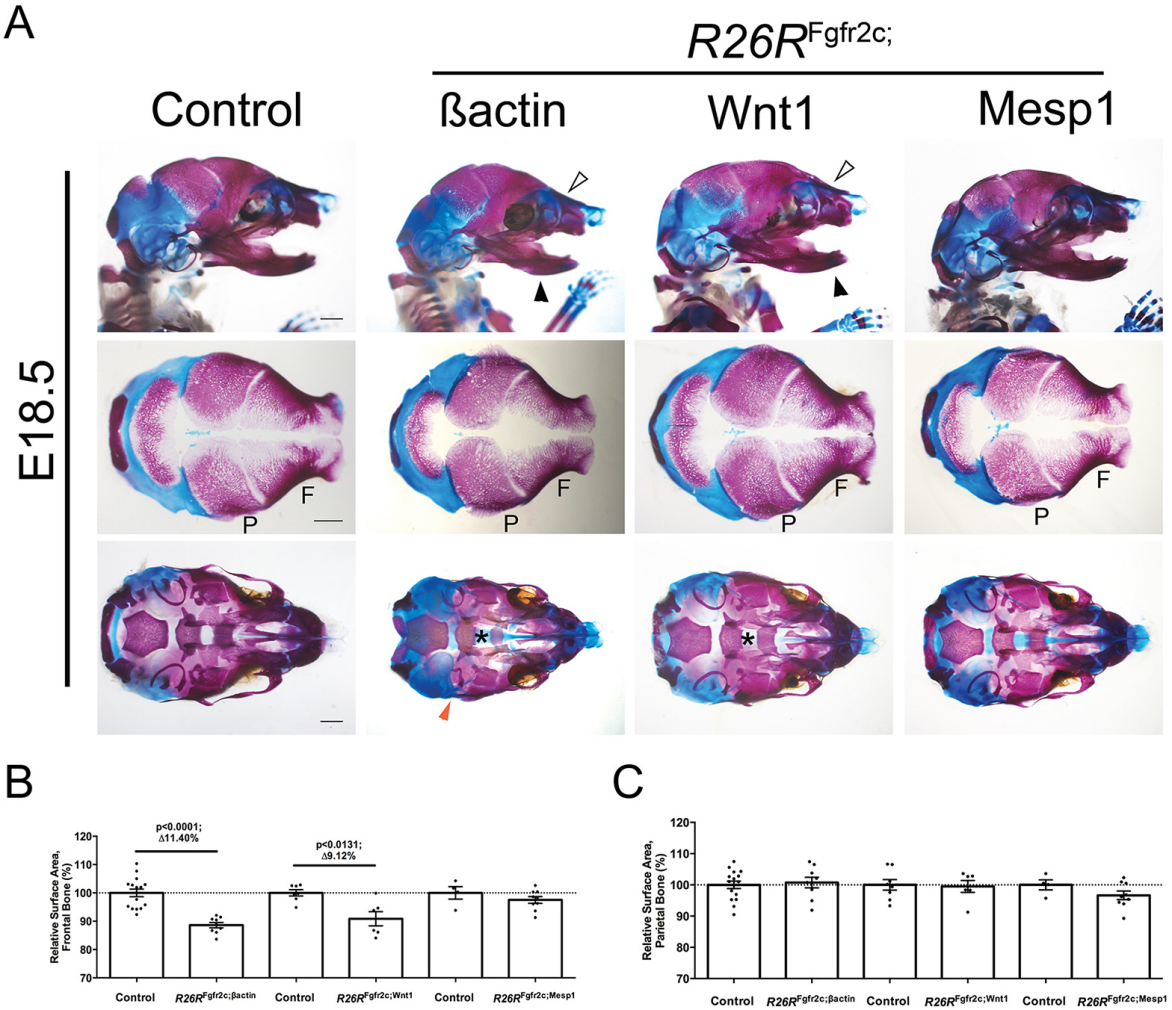
**Craniofacial hypoplasia of the NCC lineage in E18.5 mutants.** (A) *R26R*^Fgfr2c;βact^ and *R26R*^Fgfr2c;Wnt1^ embryos display reductions of the nasal bones and mandibles (white and black arrowheads, respectively). Other features include an overt cleft palate (asterisk) in *R26R*^Fgfr2c;βact^, but only a partially penetrant cleft palate (asterisk) in *R26R*^Fgfr2c;Wnt1^ embryos. Tympanic ring hypoplasia is exclusive to *R26R*^Fgfr2c;βact^ embryos (red arrowhead). No apparent phenotype was observed in *R26R*^Fgfr2c;Mesp1^ mice. (B,C) Quantitative analysis of the frontal and parietal bones. Significant reductions of the frontal bone were observed in both *R26R*^Fgfr2c;βact^ (*P*<0.0001) and *R26R*^Fgfr2c;Wnt1^ (*P*<0.0131) embryos. Statistics: Student's *t*-test with Welch's correction. F, frontal bone; P, parietal bone. Scale bars: 1 mm.

To assess which tissues are sensitive to increased FGFR2c signalling, *R26R*^Fgfr2c-flox^ mice were crossed with *βactin*^CRE^ (also known as *Actb*^CRE^) mice to drive ubiquitous *Fgfr2c* overexpression (Fig. 1A). Ubiquitous overexpression of the *R26R*^Fgfr2c^ allele (i.e. *R26R*^Fgfr2c-flox^; *βactin*^CRE^, abbreviated to *R26R*^Fgfr2c;βact^) led to a total upregulation of *Fgfr2c* transcripts close to 2-fold (*n*=3) as shown by quantitative PCR (Fig. S1A) at embryonic day (E)12.5. Transgenic FGFR2c-V5 protein expression was validated at E12.5 by immunoblotting showing transgenic protein in the CRE-positive animals only (Fig. S1B). Subsequently, *R26R*^Fgfr2c;βact^ embryos were examined at E18.5 for their size [head length and crown-rump length (CRL)] and weight (Fig. 1C-E). Results were expressed as an average percentage change (Av.Δ %) relative to controls (*R26R*^Fgfr2c-flox^). Ubiquitous *Fgfr2c* overexpression (*n*=9) led to a significant reduction in the head length [Av.Δ 7.19%; *P*<0.0001; t(17.78)=7.74], CRL [Av.Δ 4.83%; *P*=0.0018; t(13.23)=3.90] and weight [Av.Δ 12.26%; *P*=0.0001; t(22.16)=5.39] compared with that of controls (*R26R*^Fgfr2c-flox^; *n*=17) (Fig. 1C-E). Additionally, *R26R*^Fgfr2c;βact^ mice displayed microtia characterised by a smaller or absent pinna (*n*=6/6) (Fig. 1B). To assess whether decreased size was caused by generalised defects of the whole skeleton, we analysed limb length as a proxy for this (Fig. S2). No difference in the size of the limbs (*n*=13) was detected, suggesting that the overall size reduction was mostly due to reduced head size. Whole-mount skeletal staining of the head showed that mutants had craniofacial dysmorphology, showing notable disruptions to the tympanic ring of the middle ear (*n*=9/10) and a cleft palate (*n*=4/10) (Fig. 2A). As different parts of the calvaria are derived from either the neural crest (NC) or mesodermal lineage (Yoshida et al., 2008), *R26R*^Fgfr2c-flox^ animals were subsequently crossed with *Wnt1*^CRE^ or *Mesp1*^CRE^ to ask which cell types are responsible for the observed phenotypes, and to eliminate any potential ectopic effects generated by the ubiquitous *βactin*^CRE^ line. To assess the promoter activity of the different CRE lines specifically in calvarial tissues, we investigated conditional fluorescent alleles (i.e. mTmG and YFP). We found that expression was robust and specific for all CRE lines (Fig. S3). *R26R*^Fgfr2c;Wnt1^ embryos (*n*=6) showed a significant decrease in weight [Av.Δ12.26%; *P*=0.0028; t(9.23)=4.05], whereas head length and CRL were not significantly reduced (Fig. 1C-E). Moreover, the clefting phenotype was heterogeneous in its severity: one of six embryos exhibited an overt cleft, whilst three of six displayed palatal shelf hypoplasia (data not shown) (Fig. 2A). In contrast to *R26R*^Fgfr2c;βact^ mice, middle (tympanic ring) or outer (pinna) ear defects were absent in this cohort of embryos (*n*=0/6) (Fig. 2A). In *R26R*^Fgfr2c;Mesp1^, no defects were present in the ear or palate (*n*=0/9) (Fig. 2A), and no significant effects on size were seen in embryos upon *Fgfr2c* overexpression in the mesoderm using *Mesp1*^CRE^ (*n*=9) compared with controls (*n*=4) (Fig. 1C-E). Together, ubiquitous and conditional *Fgfr2c* overexpression in the NC lineage stunts overall growth, resembling the phenotypic effect of *Fgfr2c*^null^ knockout mice. Additionally, *Fgfr2c* overexpression mutants display microtia and cleft palate, of which the latter is also found in *Fgfr2c*^C342Y^ mutants, but has not been identified in *Fgfr2c*^null^ mice.

### *Fgfr2c* overexpression causes anterior, NC-derived bone hypoplasia

As all observed phenotypic features in *R26R^Fgfr2c^* mice seem to affect tissues in the head, we assessed the craniofacial morphology in more detail at E18.5 using Alcian Blue/Alizarin Red whole-mount skeletal staining, and quantified the size and shape of individual craniofacial bones. The results were expressed as an average percentage change in the mutants relative to the controls (*R26R*^Fgfr2c-floxed^), normalised to 100% (Av.Δ %). Frontal bones of *R26R*^Fgfr2c;βact^ skulls were significantly smaller [Av.Δ 11.4%; *P*<0.0001; t(22.98)=6.99; *n*=9] (Fig. 2B,C), as were other NC derivatives, such as nasal bone [Av.Δ 12.90%; *P*<0.0001; t(22.06)=7.91; *n*=10] and mandible [Av.Δ 5.77%; *P*<0.0001; t(20.64)=7.30; *n*=9] (Fig. S4). No significant reduction was observed in the mesoderm-derived parietal bone. Similarly, *R26R*^Fgfr2c;Wnt1^ embryos followed a comparable trend, with significant decreases in NC derivatives: frontal bone [Av.Δ 9.12%; *P*=0.0131; t(6.89)=3.32; *n*=6], nasal bone [Av.Δ 7.91%; *P*=0.0024; t(9.077)=4.17; *n*=6] and mandible [Av.Δ 3.71%; *P*=0.0007; t(9.96)=4.82; *n*=6]. Again, no size difference was seen in the mesoderm-derived parietal bone (*n*=6). As expected, in *R26R*^Fgfr2c;Mesp1^ mice, calvarial bone sizes were unaffected. In summary, *Fgfr2c* overexpression causes bone hypoplasia of anterior, NC-derived bones, explaining the overall reduction of craniofacial dimensions.

### *Fgfr2c* overexpression does not cause coronal craniosynostosis

**Fig. 3.**
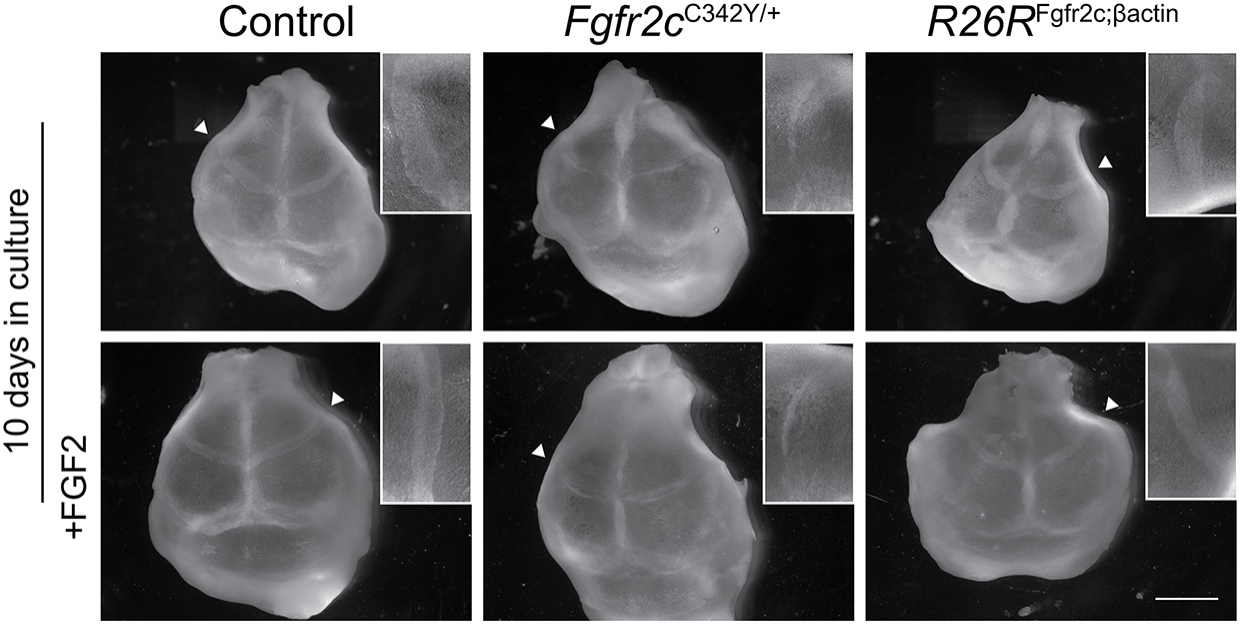
**Calvarial explant culture of *Fgfr2c*^C342Y^ and *R26R*^Fgfr2c;βact^ mutants.** Whole calvaria after 10 days in culture show partial fusion in *Fgfr2c*^C342Y^ heterozygous skulls (arrowhead, inset), whereas in *R26R*^Fgfr2c;βact^ calvaria, the coronal suture appears patent, resembling the control. Calvaria cultured with the addition of FGF2 ligand are undistinguishable from those without FGF2. Insets are a magnified image of the representative coronal suture in the respective explants. Scale bar: 1 mm.

**Fig. 4.**
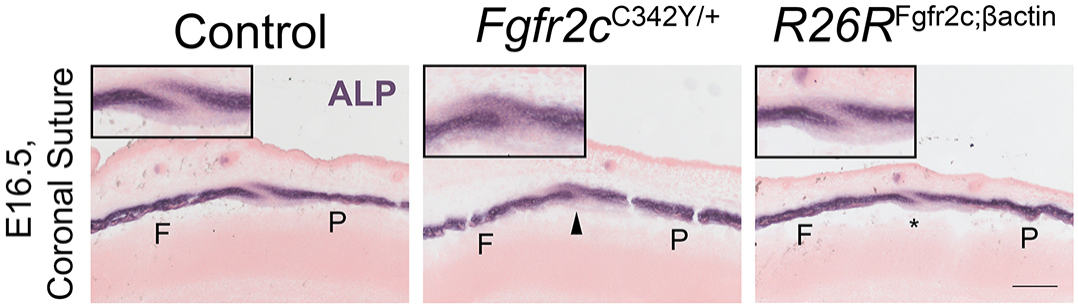
**ALP activity in the coronal suture.** *Fgfr2c*^C342Y^ and *R26R*^Fgfr2c;βact^ have dissimilar osteoblast activity in the E16.5 coronal suture. Increased appositional growth of *Fgfr2c*^C342Y^ sutures (arrowhead) and ectopic expression of ALP in sutural mesenchyme precedes synostosis; *R26R*^Fgfr2c;βact^ sutures (asterisk) resemble controls. Insets are a magnified crop of the suture. ALP, alkaline phosphatase; F, frontal bone; P, parietal bone. Scale bar: 200 μm.

Coronal synostosis is a hallmark of Crouzon and Pfeiffer syndromes, and can be observed in the *Fgfr2c*^C342Y^ mouse model as well as in *Fgfr2c*^null^ mice. Although subtle changes in the *Fgfr2c^C342Y^* heterozygous coronal suture morphology are visible from E17.5, full fusion of frontal and parietal bones is not visible until 3 weeks after birth. Owing to the presence of a cleft palate in *R26R*^Fgfr2c;βact^ embryos, mice do not survive after birth, making it impossible to assess the postnatal synostosis phenotype. *Ex vivo* explant cultures were adopted to overcome this problem. Calvarial explant cultures in our laboratory routinely show that coronal synostosis can be achieved *in vitro* after 1-2 weeks of culture in *Fgfr2c*^C342Y^ heterozygote E17.5 explants. In *R26R*^Fgfr2c;βact^ calvaria, coronal synostosis was not observed (*n*=6), similar to WT and *R26R*^Fgfr2c-floxed^ controls (*n*=7). At the same time, *Fgfr2c*^C342Y^ heterozygous coronal sutures are reduced in size and signs of suture fusion can be observed after 15 days in culture (Fig. 3)*.* Additionally, we examined the effect of FGFR2 perturbation on osteoblast maturation at a pre-synostosis embryonic stage (E16.5) by analysing alkaline phosphatase (ALP) activity. In all cases, *Fgfr2c*^C342Y^ heterozygous sutures display increased suture overlap and ectopic ALP in the sutural mesenchyme (*n*=3), whilst *R26R*^Fgfr2c;βact^ frontal and parietal bones are spaced normally and resemble controls (*n*=3) (Fig. 4). These data imply that FGFR2c overexpression does not cause coronal craniosynostosis and does not mimic FGFR2 activation in the *Fgfr2c*^C342Y^ suture.

### *Fgfr2c* overexpression and *Fgfr2c*^C342Y^ mutation activate MAPK/ERK signalling in the suture

**Fig. 5.**
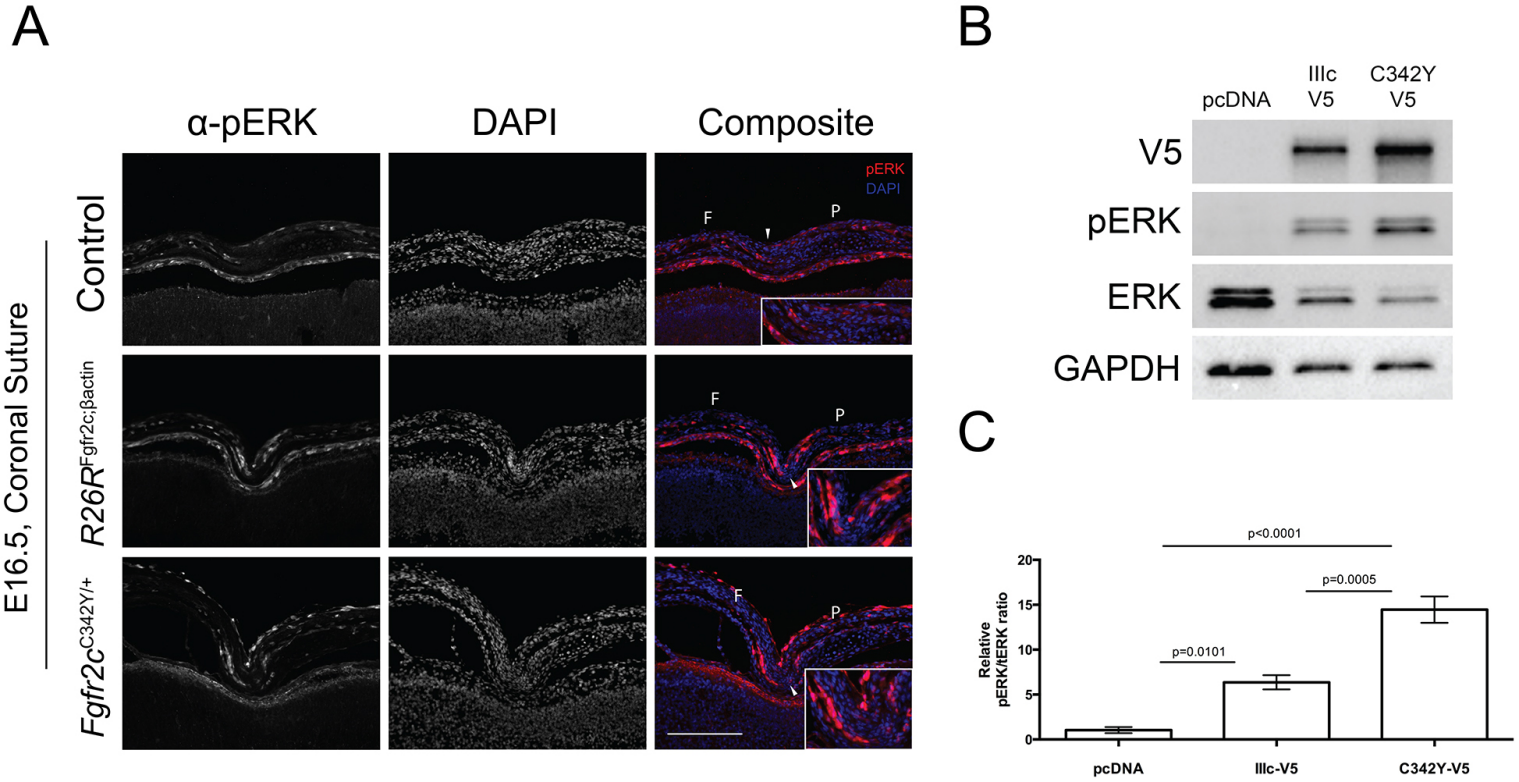
***Fgfr2c* overexpression causes upregulation of pERK *in vivo* and *in vitro.*** (A) Both *Fgfr2c*^C342Y^ and *R26R*^Fgfr2c;βact^ mutants have upregulated pERK (red) in osteogenic fronts of the frontal and parietal bones compared with controls *in vivo*. White arrowheads mark the location of the coronal suture. Insets are a cropped image of the coronal suture. (B) Western blot to demonstrate upregulation of pERK upon cellular transfection of pFGFR2c(WT)V5 (encoding the WT FGFR2-IIIc isoform) and pFGFR2c(C342Y)V5 (encoding the mutated receptor) plasmids in HEK293T cells. (C) Relative pERK:tERK ratio quantified by densitometry of transfected HEK293T cells. Statistics: one-way ANOVA with Tukey post hoc. Error bars: s.e.m. F, frontal bone; P, parietal bone. Scale bar: 200 μm.

To assess whether *Fgfr2c* overexpression leads to a similar activation of the MAPK/ERK pathway as found in the *Fgfr2c*^C342Y^ mouse, we looked at levels of phosphorylated (p)ERK in the suture *in vivo* and *in vitro*. We sought to visualise the expression of pERK in E16.5 coronal sutures using immunohistochemistry at a stage at which the sutures were morphologically similar, i.e. prior to the onset of synostosis (*R26R*^Fgfr2c;βact^, *n*=3; *Fgfr2c*^C342Y/+^, *n*=4; control, *n*=4). Interestingly, both *Fgfr2c*^C342Y/+^ and *R26R*^Fgfr2c;βact^ mutants showed upregulated pERK in the osteogenic fronts of frontal and parietal bones flanking the suture (Fig. 5A). We also modelled pERK activity *in vitro* using the human embryonic kidney (HEK293T) cell line (*n*=4 independent transfections). pERK1 and pERK2 (also known as pMAPK3 and pMAPK1, respectively) were activated upon transfection of pFGFR2c(WT)V5 (encoding the WT FGFR2-IIIc isoform) and pFGFR2c(C342Y)V5 (encoding the mutated receptor) relative to mock transfected cells (Fig. 5B). Quantification of the blots using densitometry revealed significant upregulation of pERK in both FGFR2cV5 transfected conditions (Fig. 5C). Specifically, there was significantly more pERK activity in the C342Y-V5 (*P*<0.0001) and WT-V5 (*P*=0.0101) transfected cells relative to the pcDNA3.1 control cells, as expected from the western blot results. Cells transfected with C342Y-V5 had an increased pERK output, by 8.1 units, compared with that of the WT-V5 transfected group (*P*=0.0005), likely due to the constitutive activation of the mutant receptor. Although both models show an activated RAS-MAPK pathway in the suture, the fact that only *Fgfr2c*^C342Y^ activation results in craniosynostosis suggests that FGFR2c overexpression cascade activation is functionally distinct from that of the mutant receptor.

### Introduction of the *Fgfr2c* overexpression allele into *Fgfr2c*^C342Y^ mice delays craniosynostosis

**Fig. 6.**
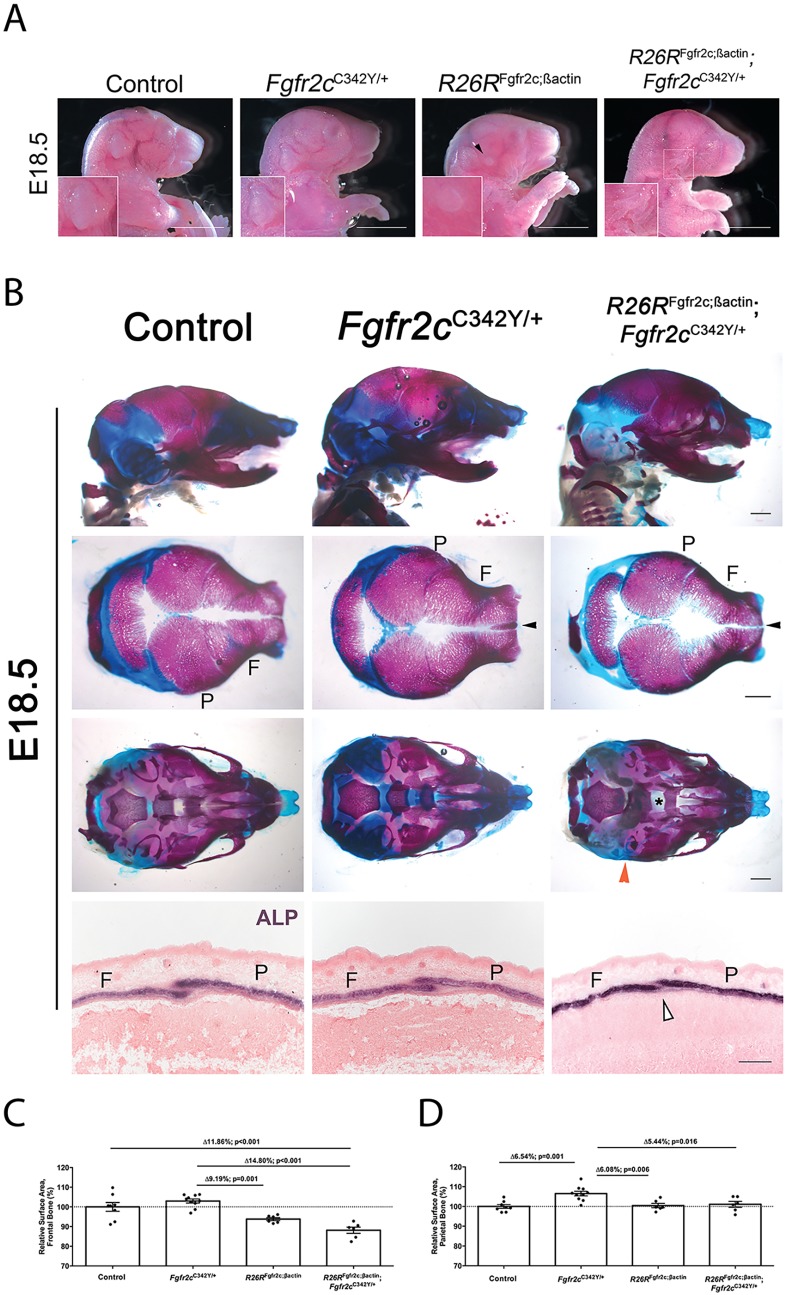
**Phenotypic analysis of *R26R*^Fgfr2c;βact^; *Fgfr2c*^C342Y^ double mutants at E18.5.** (A) *R26R*^Fgfr2c;βact^; *Fgfr2c*^C342Y^ have a more severe phenotype of the external ear (inset) compared with *R26R*^Fgfr2c;βact^ (arrowhead). Insets are magnified representations of the ear phenotype. (B) Craniofacial phenotype of the *R26R*^Fgfr2c;βact^; *Fgfr2c*^C342Y^. Wormian bone rescue in *R26R*^Fgfr2c;βact^; *Fgfr2c*^C342Y^ and a wider frontal posterior interfrontal suture are observed (black arrowheads). Other features include severe tympanic ring hypoplasia (red arrowhead) and cleft palate (asterisk). Bottom row is ALP assays, in which partial sparing of the coronal suture in *R26R*^Fgfr2c;βact^; *Fgfr2c*^C342Y^ is observed. White arrowhead indicates the region affected. (C,D) Quantitative analysis of the calvarial bones. Significant size reduction of the frontal and parietal bones in *R26R*^Fgfr2c;βact^; *Fgfr2c*^C342Y^ compared with *Fgfr2c*^C342Y^ (*P*<0.001 and *P*=0.016, respectively). Statistics: one-way ANOVA with Tukey's post hoc. ALP, alkaline phosphatase; F, frontal bone; P, parietal bone. Scale bars: 1 mm.

As FGFR2c overexpression does not cause coronal suture synostosis, we hypothesised that the addition of this allele to the *Fgfr2c*^C342Y^ mice would modify the craniofacial phenotype. To assess the impact of the overexpression allele, we generated a double mutant (i.e. *R26R*^Fgfr2c;βact^; *Fgfr2c*^C342Y^) and performed quantitative analysis on the calvaria as before (Fig. 6). The most apparent external anomaly resulting from *Fgfr2c* overexpression alone was microtia. This was exacerbated in double mutants, in which anotia was present in 90% of mice (Fig. 6A) (*n*=10/11). External ear development was normal in all *Fgfr2c*^C324Y^ single mutants (*n*=15). Double mutants showed cleft palate with an increased penetrance (*n*=7/7). Examination of the craniofacial skeleton reveals partial rescue of the Crouzon phenotype: an ectopic interfrontal Wormian bone is characteristic of *Fgfr2c*^C342Y^ heterozygotes (*n*=6/7), and these were generally smaller in double mutants (*n*=4/5), combined with an enlarged widening of the posterior interfrontal suture (Fig. 6B). In general, quantitative analyses of calvarial bones show that *R26R*^Fgfr2c;βact^; *Fgfr2c*^C342Y^ frontal bones were smaller than those of controls (Av.Δ 11.86%; *P*<0.001; control, *n*=8, double mutant, *n*=6) (Fig. 6C). Significance size reduction was present when *R26R*^Fgfr2c;βact^ frontal bones (*n*=6) were compared with *Fgfr2c*^C342Y^ frontal bones (*n*=10) (Av.Δ 9.19%; *P*=0.001). Also, there was a significant decrease in frontal bone size in *R26R*^Fgfr2c;βact^; *Fgfr2c*^C342Y^ compared with *Fgfr2c*^C342Y^ (Av.Δ 14.80%; *P*<0.001). Quantitative analysis of the parietal bones indicated a significant increase in the parietal bone of *Fgfr2c*^C342Y^ (*n*=10) compared with all other genotypes (control, Av.Δ 6.54%, *P*=0.001, *n*=8; *R26R*^Fgfr2c;βact^, Av.Δ 6.08%, *P*=0.006, *n*=6; *R26R*^Fgfr2c;βact^; *Fgfr2c*^C342Y^, Av.Δ 5.44%, *P*=0.016, *n*=6) (Fig. 6D). Most strikingly, coronal sutures in double mutants appeared more patent than those in *Fgfr2c*^C342Y^ alone. Frontal and parietal bone overlap was decreased in *R26R*^Fgfr2c;βact^; *Fgfr2c*^C342Y^ coronal sutures, as shown by Alizarin Red staining and ALP activity (Fig. 6B) (control, *n*=2; *Fgfr2c^C342Y^*, *n*=2; *R26R*^Fgfr2c;βact^; *Fgfr2c*^C342Y^, *n*=3). However, the observed ectopic ALP in the sutural mesenchyme of *Fgfr2c*^C342Y^ animals (Fig. 4) was not decreased in the majority of double mutant sutures (Fig. 6B). This suggests that the overexpression allele is potentially only delaying the synostosis process, possibly through calvarial bone hypoplasia caused by decreased osteogenic differentiation or increased mesenchymal proliferation.

### Increased proliferation in *R26R*^Fgfr2c;βact^; *Fgfr2c*^C342Y^ double mutants

**Fig. 7.**
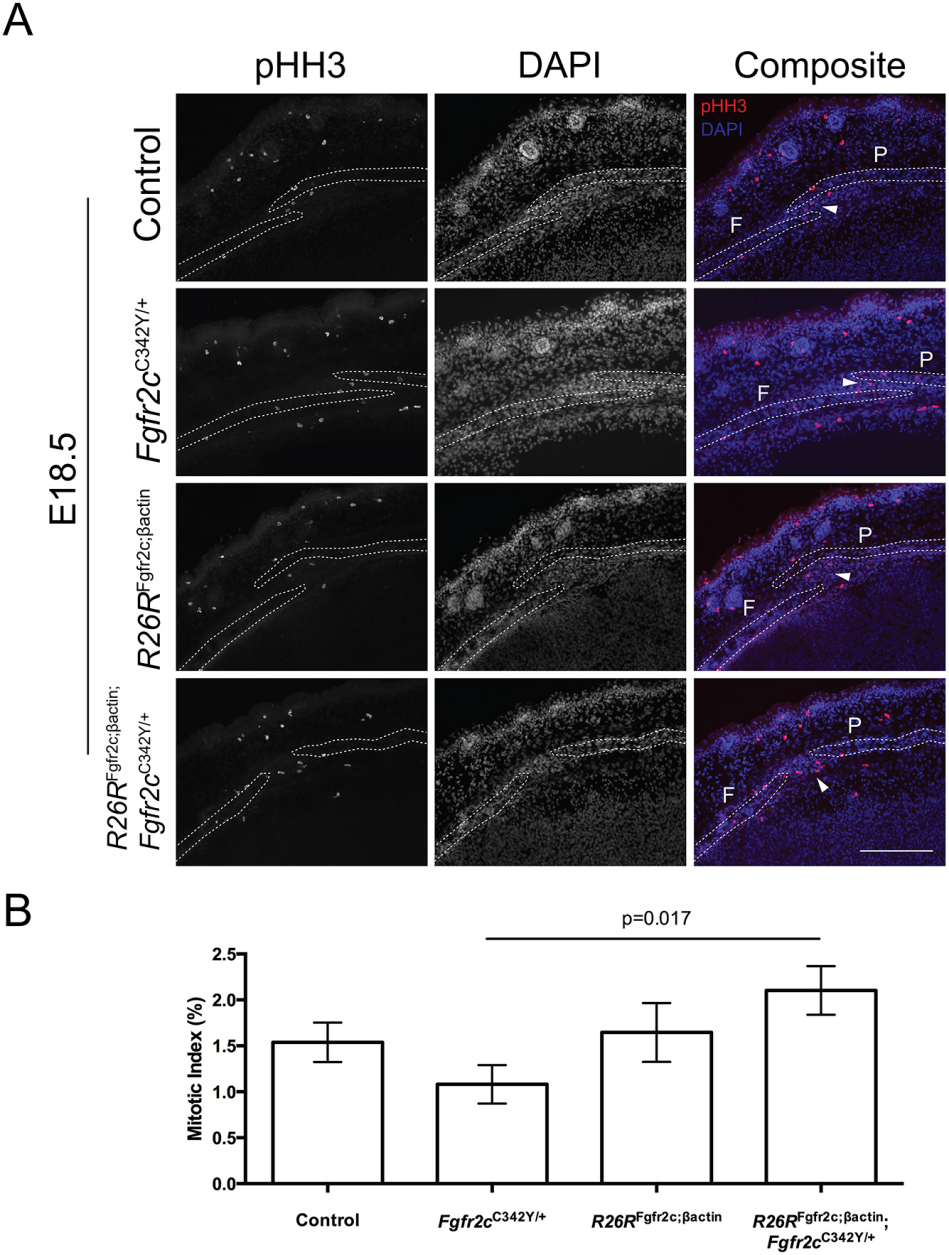
**Analysis of proliferation by pHH3 immunohistochemistry.** (A) pHH3 staining (red) in sagittal sections through the coronal suture of *R26R*^Fgfr2c;βact^ and *Fgfr2c*^C342Y^ single mutants. *R26R*^Fgfr2c;βact^; *Fgfr2c*^C342Y^ double mutants show an apparent increased number of proliferative cells in the suture mesenchyme (arrowheads). (B) Quantification of the proliferation assay shows a statistically significant increase (*P*<0.05) in *R26R*^Fgfr2c;βact^; *Fgfr2c*^C342Y^ double mutants alone compared with *Fgfr2c*^C342Y^. F, frontal bone; P, parietal bone. Scale bar: 200 µm.

To test the hypothesis that altered proliferation in the suture underlies the delayed onset of craniosynstosis in *R26R*^Fgfr2c;βact^; *Fgfr2c*^C342Y^ double mutants, we performed phosphohistone-3 (pHH3) immunohistochemistry to assess mitotic index (Fig. 7A). Although levels of proliferation were unchanged in the periosteum overlying the coronal suture, a statistically significant (*P*<0.05) increase was detected in the sutural mesenchyme of double mutants compared with *Fgfr2c*^C342Y^ mutants alone (Fig. 7B). This implies that the addition of the *R26R*^Fgfr2c;βact^ allele increases sutural proliferation, which, in turn, delays osteogenic differentiation. This leads to bone hypoplasia and rescues the craniosynostosis phenotype.

## DISCUSSION

The paradox of FGFR2c signalling was first implicated when it was found that craniosynostosis can be caused by deletion of the IIIc isoform (*Fgfr2c*^null^), as well as by introducing an activating mutation linked to human Crouzon syndrome (*Fgfr2c*^C342Y^) (Eswarakumar et al., 2002, 2004). *Fgfr2c*^C342Y^ heterozygotes display an early postnatal craniosynostosis phenotype, whereas *Fgfr2c*^null^ mice show late postnatal onset (Eswarakumar et al., 2004, 2002). We sought to increase our understanding of FGFR2c signalling misregulation through *Fgfr2c* overexpression and comparison with *Fgfr2c*^C342Y^. Our data show that global *Fgfr2c* overexpression yields craniofacial hypoplasia, microtia and cleft palate. Strikingly, this cohort of mutants did not develop coronal synostosis, as opposed to *Fgfr2c*^C342Y^ and *Fgfr2c*^null^ mutants.

There are no reports of external ear defects related to human Crouzon syndrome or in *Fgfr2c*^C342Y^mice. This study describes microtia with hypoplasia of the tympanic ring in *R26R*^Fgfr2c;βact^ that has similarities to lacrimo-auriculo-dento-digital (LADD) syndrome, which can be caused by mutations in *FGF10* or *FGFR2* (Rohmann et al., 2006). However, the external ear phenotype observed in the *R26R*^Fgfr2c;βact^ is more likely to be a consequence of ectopic expression of FGFR2c in the ectoderm, potentially activated in an autocrine way by ectodermal FGF ligands with a high affinity for the IIIc isoform (Wright and Mansour, 2003). The lack of an external ear phenotype observed in other mouse models of *Fgfr2c* supports this (Eswarakumar et al., 2002, 2004). Nonetheless, a clear hypoplastic tympanic ring was noted in *R26R*^Fgfr2c;βact^ mutants, a phenotype similar to *Fgfr2c*^null^ embryos at E18.5, implying the importance of FGFR2c in the osteogenesis of the auditory bulla (Eswarakumar et al., 2002).

Murine palatogenesis commences at E11.5 and is complete by E17.5 (Bush and Jiang, 2012). The prevalence of cleft palate in human Crouzon syndrome is less than that in Apert patients, which may be due to the mutation affecting both isoforms in the latter (Stanier and Pauws, 2012). The overt cleft phenotype in both *R26R*^Fgfr2c;βact^ and *R26R*^Fgfr2c;Wnt1^ was not fully penetrant: 40% (4/10) of *R26R*^Fgfr2c;βact^ embryos and 16% (1/6) of *R26R*^Fgfr2c;Wnt1^ embryos, respectively. As *Fgfr2b* is a major player for palatal shelf elevation, the partial penetrance implicating the IIIc isoform is less critical to this process, which is supported by the observation that *Fgfr2c*^null^ mice do not have a cleft palate phenotype (Rice et al., 2004; Eswarakumar et al., 2002). However, in the double mutants (i.e. *R26R*^Fgfr2c;βact^; *Fgfr2c*^C342Y^), the penetrance of cleft palate was 100% (*n*=7/7), indicating that the combination of the constitutively active receptor with *Fgfr2c* overexpression exacerbates the cleft phenotype, resembling the *Fgfr2c*^C342Y^ homozygotes (Peskett et al., 2017).

Our data support the conclusion that craniofacial hypoplasia is likely to be a result of conditional *Fgfr2c* overexpression in the NC lineage. A previous quantitative analysis of the adult *Fgfr2c*^C342Y^ reports smaller frontal and nasal bones, accompanied by a short anterior cranial base (Liu et al., 2013). Diminished calvarial bone volume was also reported in other mouse models for syndromic craniosynostosis, most notably in *Fgfr2*^S252W^ and *Fgfr3*^P244R^ (Muenke syndrome) (Twigg et al., 2009; Chen et al., 2003). This is consistent with our finding that hypoplasia of NC-derived bones is present in E18.5 *R26R*^Fgfr2c;βact^ mice. Instead of a specific effect on NC-derived tissues, it is also possible that ectopic expression of FGFR2c in the *R26R*^Fgfr2c;βact^ mice plays a role. As mentioned before, expression of FGFR2 in the ectoderm is the likely explanation for the microtia phenotype. Similarly, ectopic expression in any other non-NC cells, or increased expression of FGFR2 in NC and mesoderm concomitantly during embryonic development of the head might contribute to the different craniofacial phenotype found in *R26R*^Fgfr2c;βact^ mice compared with *R26R*^Fgfr2c;Wnt1^ and *R26R*^Fgfr2c;Mesp1^ mice. Interestingly, *Fgfr2c*^C342Y^ embryos at E18.5 do not show similar signs of hypoplasia at this stage. This is likely due to the nature of the C342Y mutation, which plays different roles in both early and late stages of development (Liu et al., 2013; Mansukhani et al., 2000). The C342Y mutation favours premature osteoblast commitment, but inhibits bone mineralisation and facilitates cellular apoptosis during late gestation (Liu et al., 2013; Mansukhani et al., 2000; Rice et al., 1999). Despite similarities observed in the anterior bone phenotype of *Fgfr2c*^C342Y^ and *R26R*^Fgfr2c;βact^, it is likely that the mechanism behind the phenotype is different. We propose that the hypoplasia found in *R26R*^Fgfr2c;βact^ mutants is mainly a result of insufficient osteogenesis, but we cannot exclude an ectopic effect at this stage.

A major difference between the *R26R*^Fgfr2c;βact^ mice and *Fgfr2c*^C342Y^ mutants is the absence of coronal synostosis in the overexpression model; *R26R*^Fgfr2c;βact^ mutants do not mimic the coronal synostosis phenotype of *Fgfr2c*^null^ mice. It is likely that the overall signalling disruption by receptor overexpression is less extreme than that of complete signalling removal (*Fgfr2c*^null^) or a constitutively active receptor (*Fgfr2c*^C342Y^). This is reflected by the relatively subtle phenotypic spectrum, even with ubiquitous receptor overexpression under the control of the *βactin* promoter (*R26R*^Fgfr2c;βact^). MAPK/ERK signalling is confined within a specific spatial domain along the membranous bones and the osteogenic fronts of the coronal suture. This was expected, as the expression of Spry gene downstream targets coincides with periosteoblast cells known to be expressing *Fgfr2* (Deckelbaum et al., 2005; Johnson et al., 2000). Also, the relevant FGF ligands are confined to the osteogenic front. For example, *Fgf18* and *Fgf20* transcripts were detected in the osteogenic fronts of the coronal suture, coinciding with those genes involved in FGFR2 signalling, pointing towards potential autocrine interactions (Hajihosseini and Heath, 2002; Ornitz and Itoh, 2015). However, owing to the nature of ubiquitous overexpression and the morphogenic nature of FGFs, we expected ectopic expression to affect the *R26R*^Fgfr2c;βact^ suture mesenchyme. The lack of phenotype in these overexpressing mutants suggests that, although pERK is upregulated in both *Fgfr2c*^C342Y^ and *R26R*^Fgfr2c;βact^, only *Fgfr2c*^C342Y^ sutures show increased differentiation as revealed by increased levels of ALP, hence the craniosynostosis phenotype. Together, these results suggest that augmentation of MAPK/ERK signalling in the suture alone is not sufficient to derive a coronal synostosis phenotype in the craniofacial skeleton (Eswarakumar et al., 2004; Pfaff et al., 2016; Li et al., 2007).

Phenotypic rescue of the coronal suture could be generally achieved at the level of the receptor, such as by uncoupling Frs2 or through ERK knockdown (Eswarakumar et al., 2006; Shukla et al., 2007). Ultimately, the aim is to ameliorate the Crouzon phenotype through FGFR2c signalling attenuation. We sought to address whether *Fgfr2c* overexpression is sufficient to delay ossification in *Fgfr2c*^C342Y^, given the biochemical properties reported *in vitro* (Miraoui et al., 2009; Baddoo et al., 2003)*.* Previous studies attempted to elucidate the paradoxical nature of FGFR2 signalling, where the perception of a GOF mutation leads towards signalling activity attenuation (Snyder-Warwick et al., 2010; Pfaff et al., 2016; Bagheri-Fam et al., 2015). Specifically, two studies attempted to ameliorate phenotypes caused by the C342Y allele, by introducing the C342Y allele into the *Fgfr2c*^null^ background, but yielded a more severe phenotypic spectrum (Pfaff et al., 2016; Bagheri-Fam et al., 2015). Our data suggest that the *Fgfr2c* overexpression allele was sufficient to delay synostosis. In particular, the extent of suture overlap in *R26R*^Fgfr2c;βact^; *Fgfr2c*^C342Y^ mutants was decreased compared with control or *Fgfr2c*^C342Y^ mice. However, suture abolishment remains apparent due to the presence of the *Fgfr2c*^C342Y^ allele. As FGFR2 is critical for cell renewal, one speculation for this rescue is shifting the balance from osteoblast differentiation to proliferation through ‘scavenging activity’ of endogenous FGF ligands by excess FGFR2c. Our results show that there is a distinct increase in proliferation in the sutural mesenchyme of *R26R*^Fgfr2c;βact^; *Fgfr2c*^C342Y^ double mutants, which would indicate a shift from differentiation to proliferation, explaining the osteogenic hypoplasia and the absence of craniosynostosis. This hypothesis is also supported by *in vitro* culture experiments with mesenchymal stem cells (MSCs), as exposure to FGF2 promotes stemness in the presence of osteoblast differentiation media (Baddoo et al., 2003). Another possibility is that the addition of an extra WT *Fgfr2c* allele into the *Fgfr2c*^C342Y^ heterozygous mice disrupts the receptor turnover at the membrane and/or disturbs the dimerisation equilibrium, favouring WT homodimers over WT/C342Y heterodimers. Altogether, the delayed synostosis in double mutants suggests that *Fgfr2c* overexpression partially rescues reduced premature osteoblast commitment caused by the C342Y allele (Eswarakumar et al., 2004; Peskett et al., 2017).

We propose the FGFR2c paradox should not be based solely on the signalling amplitude but, rather, the cascade that FGFR2c transactivates. This is due to activation of TRK receptors, such as FGFR2, which triggers signal transduction through three major cascades (PI3K-AKT, RAS-MAPK and PLCγ-PKC) (Ornitz and Itoh, 2015). It was previously reported that GOF mutations in the FGFR2c receptor elicit dissimilar cascade activation to that of WT-FGFR2c (Kim et al., 2003; Miraoui et al., 2009). Miraoui et al. (2009) compared the differences in cascade activation and concluded that Apert-FGFR2-S252W transactivates the PLCγ-PKC pathway to drive osteoblast differentiation and mineralisation, whereas WT-FGFR2 predominantly signals through the RAS-MAPK pathway (Miraoui et al., 2009). Other growth factor pathways involving TRK receptor misregulation behave similarly; for example, PDGFRα, for which sustained activation of the receptor is responsible for complex craniosynostosis through PLCγ-PKC cascade (Moenning et al., 2009). Generally speaking, augmentation of RAS-MAPK signalling promotes proliferation, whilst cellular differentiation is a consequence of cascade suppression (Dinsmore and Soriano, 2018). This cellular consequence is comparable to embryonic stem cells maintaining pluripotency (Yamanaka et al., 2010). For example, in the murine palate, it is understood that proliferation of palatal mesenchyme cells is driven by activated ERK through exposure to FGFs in culture, and that its inhibition led to the downregulation of ‘stemness’ genes (Vasudevan et al., 2015). In the same study, genetic ablation of *Fgfr1* (*Fgfr1*^null^) led to ectopic osteoblast differentiation in the palatal shelves *in vivo* (Vasudevan et al., 2015)*.* Unpublished data from this laboratory (K.K.L.L., E. Pauws) also support this, as oncogenic activation of KRas (i.e. KRas-G12D), an effector of the MAPK/ERK pathway in the NC lineage (*Kras*^G12D;Wnt1^), led to severe craniofacial hypoplasia. Oncogenic KRas is known to cause terminal differentiation defects, suggesting that upregulation of MAPK/ERK signalling alone resulted in insufficient osteoblast differentiation (Tuveson et al., 2004; Haston et al., 2017). Therefore, the pathogenic mechanism for craniosynostosis cannot be attributed to RAS/MAPK misregulation alone.

In summary, this study has demonstrated that FGFR2c overexpression yields craniofacial hypoplasia without a craniosynostosis phenotype. The most striking observation is the phenotypic variation between *R26R*^Fgfr2c;βact^ and *Fgfr2c*^C342Y^, despite similarities in signalling dynamics. This implies that receptor overexpression and GOF mutations are mechanistically different, and require a different downstream interpretation to the WT. This is well characterised *in vitro* with preferential signal transduction, and our results relate these findings to an *in vivo* context. Maintaining the correct balance between proliferation and differentiation is crucial for osteogenesis and suture patency. Elucidating the role of FGFR2c signalling will improve the understanding of normal craniofacial development and its related pathologies, while providing a framework for the development of novel therapeutic strategies.

## MATERIALS AND METHODS

### Animals

#### *Fgfr2c* overexpression mouse [Gt(ROSA)26Sor^tm1(Fgfr2-IIIc)Pauw^; also known as *R26R^Fgfr2c-flox^*; MGI:6150825]

To target the *Rosa26* genomic locus [Gt(ROSA)26^tm1Sor^], an *Fgfr2c* complementary DNA (cDNA) expression construct containing a V5 epitope on the C-terminal end of the protein was inserted into a targeting vector carrying the loxP-PGK-neo-tPa-loxP cassette (Soriano, 1999), followed by homologous recombination and the creation of chimera. Germline mice carrying the *R26R*^Fgfr2c-flox^ allele were backcrossed onto a CD1 background and maintained as homozygotes. To generate *Fgfr2c* overexpressing mutants, mice carrying the *R26R*^Fgfr2c-flox^ allele were crossed with animals carrying a CRE recombinase allele (*βactin*^CRE^, *Wnt1*^CRE^ or *Mesp1*^CRE^). Embryos were genotyped for CRE recombinase and loxP excision. Genotyping primers are available upon request.

#### Crouzon mouse model (*Fgfr2*^tm4Lni^; also known as *Fgfr2c*^C342Y^; MGI:3053095)

*Fgfr2c*^C342Y^ were re-derived through the European Mouse Mutant Archive (EMMA) at MRC Harwell as previously described (Peskett et al., 2017).

Generation of double mutants was performed by crossing *R26R*^Fgfr2c-flox^ animals with *Fgfr2c*^C342Y^ heterozygotes, followed by genotyping for both alleles. *Fgfr2c*^C342Y^; *R26R*^Fgfr2c-flox^ animals were subsequently crossed with *βactin*^CRE^ to generate ubiquitous overexpression in the Crouzon mouse (i.e. *R26R*^Fgfr2c;βact^; *Fgfr2c*^C342Y^).

*Wnt1*^CRE^; *R26R*^YFP^ was as previously described (Freem et al., 2010). CRE recombinase is driven under the control of the *Wnt1* promoter to generate conditional expression in the NC cell lineage. *Wnt1*^CRE^ mice were crossed with *R26R*^YFP/YFP^ reporter to generate *Wnt1*^CRE/+^; *R26R*^YFP/+^ offspring. Cells positive with the *CRE* allele will express the reporter protein, thus labelling cells from the NC cell lineage.

*R26R*^mTmG/mTmG^ were as previously described (Muzumdar et al., 2007). The *mTmG* allele was crossed with *R26R*^Fgfr2cV5/Fgfr2cV5^ to generate a double-conditional mouse that overexpresses both *mTmG* and *Fgfr2cV5* (*R26R*^mTmG/+^; *R26R*^Fgfr2cV5/+^) upon CRE deletion.

All animal procedures were performed in accordance with the UK Animals (Scientific Procedures) Act 1986 (project license number 70/8817). Animals were maintained by UCL Biological Services.

### Cells

HEK293T cells were cultured in minimum essential medium (MEM) alpha culture medium (Thermo Fisher Scientific) supplemented with 10% fetal bovine serum (FBS) and penicillin-streptomycin. Cells were transfected with pcDNA3.1 (Invitrogen) plasmids containing an *Fgfr2c* ORF (WT and C342Y) with a V5 tag on the C-terminal end, as well as control pcDNA3.1 without insert, when they had reached 50% confluence. A cocktail of Optimem culture medium (Gibco), FuGENE transfection reagent (Promega) and plasmid DNA was added to each well prior to 48 h incubation at 37°C, 5% CO_2_.

### Immunoblots

E12.5 embryos were dissected under ice-cold PBS and homogenised in RIPA buffer [150 mM NaCl, 1% Triton X-100 (Fisher), 0.5% sodium deoxycholate (Sigma-Aldrich), 0.1% SDS (Sigma-Aldrich), 50 mM Tris-pH 8.0 (Fisher)] with MINI complete protease inhibitor cocktail (Sigma-Aldrich) and centrifuged to obtain the lysates. Protein concentration was determined using Bradford reagent (Bio-Rad) and spectrophotometry. A special cell lysis buffer [50 mM pH7.6 Tris-Base; 150 mM NaCl, 1% Triton X-100, 0.02% sodium azide, 1 mM MINI protease inhibitor (Roche), 1 mM sodium orthovanadate, 25 mM sodium fluoride] was adopted for cells. Lysates were resolved, transferred and blotted using standard protocols. Anti-V5 antibody (Invitrogen) was used at a concentration of 1:1000 for E12.5 embryos and 1:3000 for cell lysates. Anti-pERK (1:2000, Cell Signaling Technology) and anti-total (t)ERK (1:2000, Cell Signaling Technology) were used to determine pathway activity. pERK blots were quantified by densitometry using FIJI software (Schindelin et al., 2012), and results expressed as the relative pERK:tERK ratio.

### Quantitative RT-qPCR

RNA was extracted using the Trizol method (Invitrogen). Any genomic DNA was subsequently eliminated using the DNA-free DNA Removal Kit (Ambion) prior to the reverse transcription reaction. A QuantiTect Reverse Transcription Kit (Qiagen) was used for cDNA synthesis. cDNA was used for RT-qPCR reactions using Taqman assays (Applied Biosystems) according to the manufacturer's protocol. *Fgfr2c* assay (Applied Biosystems; Mm01269938_m1) was used to determine *Fgfr2c* overexpression. The collected dataset was analysed using the 7500 Fast Real-Time PCR System (Applied Biosystems). Amplification efficiencies were checked for target genes and controls prior to data analysis using the ΔΔCt method.

### *Ex vivo* calvarial explants

A calvarial explant protocol was performed as previously described (Lana-Elola et al., 2007). Embryos were harvested at E17.5 and dissected in PBS. The calvaria was removed from the skin and brain and cultured in Dulbecco's modified Eagle medium (DMEM, Sigma-Aldrich) supplemented with 10% FBS (Sigma-Aldrich) and penicillin-streptomycin. The medium, with or without the addition of 10 ng/ml FGF2, was refreshed every other day and cultured for 10-15 days at 37°C, 5% CO_2_. Calvaria were fixed in 90% ethanol and processed for Alizarin Red staining.

### Gross phenotypic analysis

Embryos were weighed using a fine balance. An electronic caliper (Fisher) was used to measure the CRL and head length of the embryo.

### Whole-mount skeletal staining

The procedure for whole-mount skeleton histology was as previously described (Peskett et al., 2017). E18.5 embryos were skinned, eviscerated and fixed in 90% EtOH overnight at 4°C and stained with Alcian Blue (Sigma-Aldrich) working reagent (0.01% Alcian Blue; 20% acetic acid; 80% of 75% EtOH) overnight at room temperature. Embryos were washed in 75% EtOH for a further 24 h and cleared in 1% potassium hydroxide (KOH) the following day. After sufficient clearing, 0.01% Alizarin Red (Sigma-Aldrich) working solution (0.01% Alizarin Red in 1% KOH) was added to the embryos. The embryos were stained overnight and washed in 1% KOH the following day. Graded glycerol (Sigma-Aldrich) was used to prepare samples for imaging and long-term storage in 80% glycerol.

### Quantitative analysis of the calvaria

Stained E18.5 calvaria were subjected to ‘region of interest’ (ROI) analysis using ImageJ 2.0 software (National Institutes of Health). The craniofacial skeleton was dissected for the frontal, parietal and nasal bones in 80% glycerol and flat mounted onto frosted slides (Fisher). Images were taken for surface area measurements of frontal, parietal and nasal bones using ImageJ. Two measurements were made from both hemispheres of the bone, and the results were expressed as an average value. The quantification was performed blind without knowledge of the embryo's genotype. The mandibles were quantified in the same manner with the length measured instead. Quantified data were normalised to limb length to provide an endogenous control that was unaffected by increased FGFR2c signalling (Fig. S2).

### Statistical analysis

SPSS Statistics 22 (IBM) software was used as the primary statistical package for data analysis. First, the data were tested for normality using Shapiro–Wilk test to determine the use of parametric or non-parametric tests. Independent sample Student's *t*-test with Welch's correction was used to compare the difference of means between the control and mutant groups for quantification of gross phenotype and craniofacial skeleton. One-way ANOVA with Tukey post hoc or non-parametric Kruskal–Wallis with Dunn-Bonferroni post hoc test was adopted for analysis of three or more groups. A *P*-value <0.05 was considered significant. The analysed data were plotted using Prism 6.0 software (GraphPad).

### Embryo embedding and histology

For paraffin embedding, E16.5 embryo heads were skinned and fixed in 10% formalin overnight before graded dehydration in ethanol. Embryos were cleared in analytical grade xylene (Fisher) before paraffin wax displacement in a 60°C oven. The samples were embedded and sectioned between 8 and 10 μm on the axial plane using a microtome (Leica). For frozen sections, E16.5 or E18.5 heads were embedded in OCT compound and snap frozen using the −80°C isopentane method. Samples were sectioned between 15 and 20 µm on the cryostat (Bright).

### Immunofluorescence

Paraffin sections were dewaxed in Histoclear (National Diagnostics) before graded EtOH rehydration. Antigen retrieval was executed in a decloaking chamber (BioCare Medical) at 110°C for 10 min in 10 mM sodium citrate pH 6.5 buffer. Sections were permeabilised in 0.1% PBST and blocked in 10% sheep serum (Sigma-Aldrich) and blocking buffer (0.15% glycine, 2 mg/ml bovine serum albumin in 0.1% PBST). Primary antibodies were incubated on the sections overnight in 1% sheep serum (Sigma-Aldrich) and blocking buffer. pERK (rabbit mIgG, Cell Signaling Technology) was used at 1:200, and appropriate secondary antibodies were incubated for 1 h the following day. Biotin goat-anti-rabbit Alexa Fluor 488 secondary antibodies (Dako) were used against pERK. The pERK signals were amplified using Streptavidin 555 conjugates (Life Technologies) at 1:500. Sudan Black (0.1%) was applied onto tissue sections for 5 min to quench any autofluorescence, and the sections were rinsed briefly in PBST to relieve any excess staining. Finally, tissue sections were stained in 4′,6-diamidino-2-phenylindole (DAPI; Thermo Fisher Scientific) at 1:10,000 in PBS before mounting in Mowiol mounting medium (Sigma-Aldrich).

### pHH3 immunofluorescence

Standard immunofluorescence was performed on E18.5 cryosectioned heads. Briefly, cryosections were thawed at room temperature in a humidified chamber and rehydrated in PBS before fixation in 4% paraformaldehyde (PFA), 0.1% PBST permeabilisation and blocking of non-specific binding in sheep serum as described above. Anti-pHH3 (rabbit polyclonal IgG, Millipore) was incubated overnight at a concentration of 1:100 in blocking buffer prior to Alexa Fluor 488 (Invitrogen) secondary antibody detection at a concentration of 1:250 against the host species. Further washes in 0.1% PBST were carried out prior to DAPI-PBS nuclei staining as described above. Sections were mounted in Mowiol mounting medium (Sigma-Aldrich) as described above. Quantitation of pHH3 cells was achieved using a macro written for FIJI software. The mitotic index was expressed as a percentage of the total pHH3 cells with respect to the total number of nuclei. The output was subsequently processed for statistical analysis using SPSS (IBM).

### ALP assay

Cryosectioned embryos were thawed and immediately fixed in 4% PFA before permeabilisation in 0.1% TBST. Samples were equilibrated in NTMT before developing in NBT-BCIP solution. Developed samples were counterstained with Nuclear Fast Red (Sigma-Aldrich) and mounted in Mowiol mounting medium (Sigma-Aldrich).

## Supplementary Material

Supplementary information
