# Supplementary material for: Overexpression of Fgfr2c causes craniofacial bone hypoplasia and ameliorates craniosynostosis in the Crouzon mouse
Source: Dis Model Mech. 2018 Nov 9;11(11):dmm035311. doi: 10.1242/dmm.035311 (PMC6262810; doi:10.1242/dmm.035311)
Supplement: Supplementary information [file dmm-11-035311-s1.pdf]

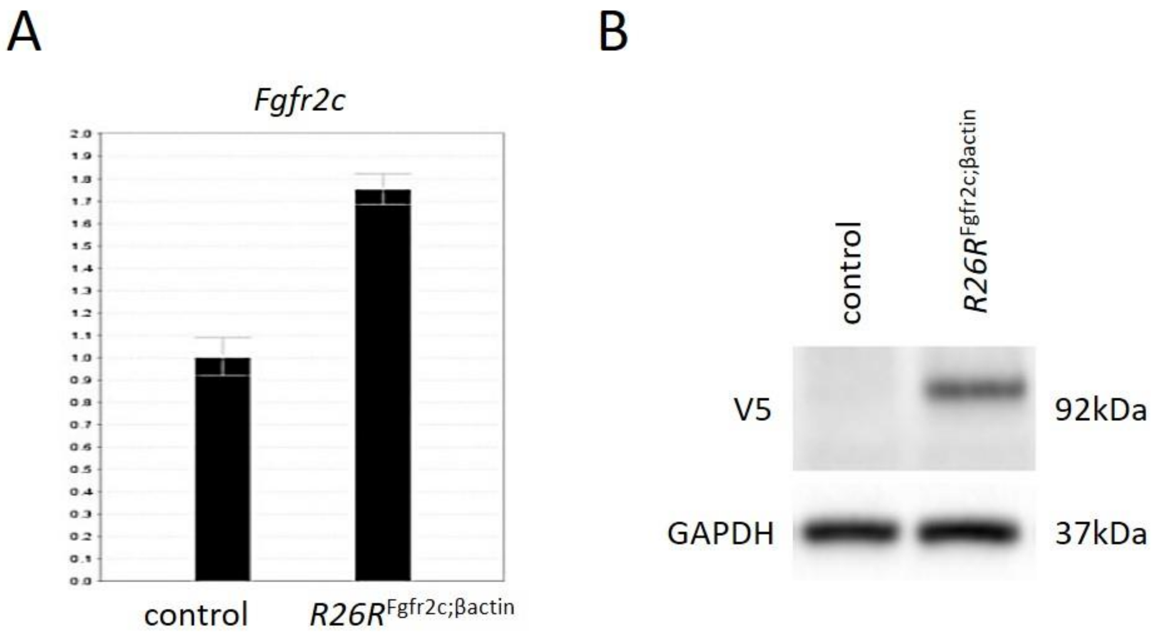

Figure S1. *Fgfr2c* RNA and FGFR2c protein expression validation.

(**A**) RT-qPCR analysis of *Fgfr2c* expression reveals upregulation of *Fgfr2c* transcripts by approximately 2-fold in *R26R*$^{\mathrm{Fgfr2c;\beta actin}}$ E12.5 embryos. (**B**) Immunoblot for the V5 epitope shows expression of the transgenic FGFR2cV5 protein in overexpression embryos only.

Disease Models & Mechanisms • Supplementary information

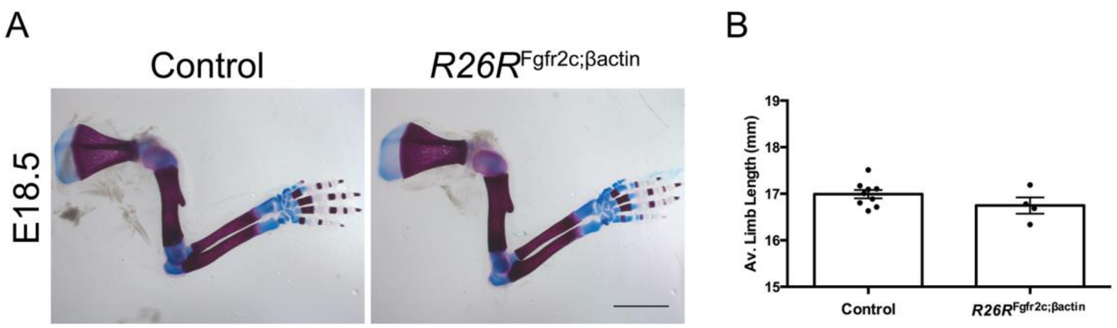

Figure S2. Quantitative analysis of *R26R*$^{Fgfr2c;\beta act}$ limb bones.

(**A**) Whole mount skeletal stained limbs of control and *R26R*$^{Fgfr2c;\beta act}$ E18.5 embryos show normal morphology.

(**B**) Quantitative analysis shows no statistically significant difference between control and mutant limb size.

Disease Models & Mechanisms • Supplementary information

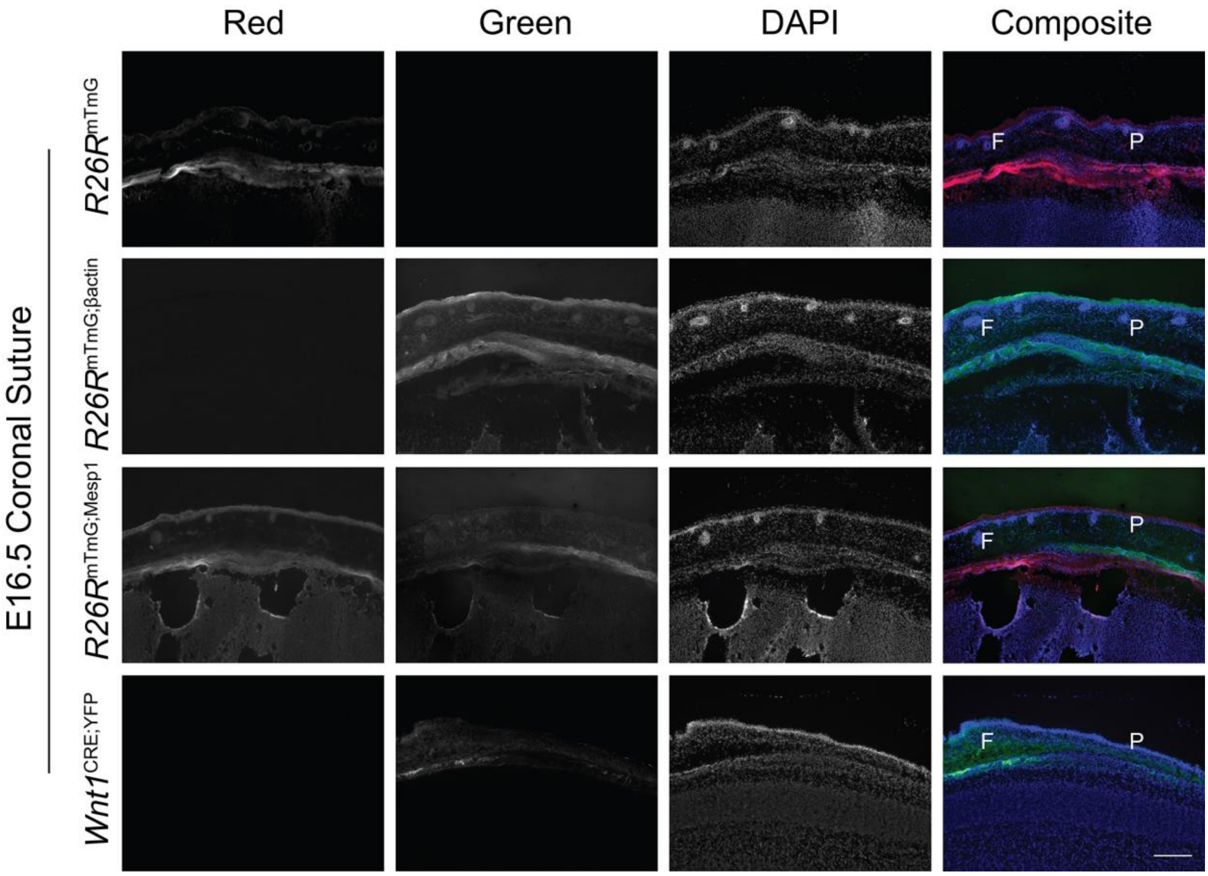

Figure S3. Reporter activity of *βactinCRE/+*, *Mesp1CRE/+* and *Wnt1CRE/+*.

eGFP (green) is only expressed in the event of CRE recombination in *R26RmTmG*, tdTomato (red) is expressed otherwise. Complete recombination is present in *R26RmTmG/+; βactinCRE/+*. Cells derived from the mesoderm are only present in the parietal bone of *R26RmTmG/+;Mesp1CRE/+*. Cells derived from the NCC lineage are only present in the frontal bone of *Wnt1CRE/+;YFP* mice. F:frontal bone, P: parietal bone; Scale bar: 200µm.

Disease Models & Mechanisms • Supplementary information

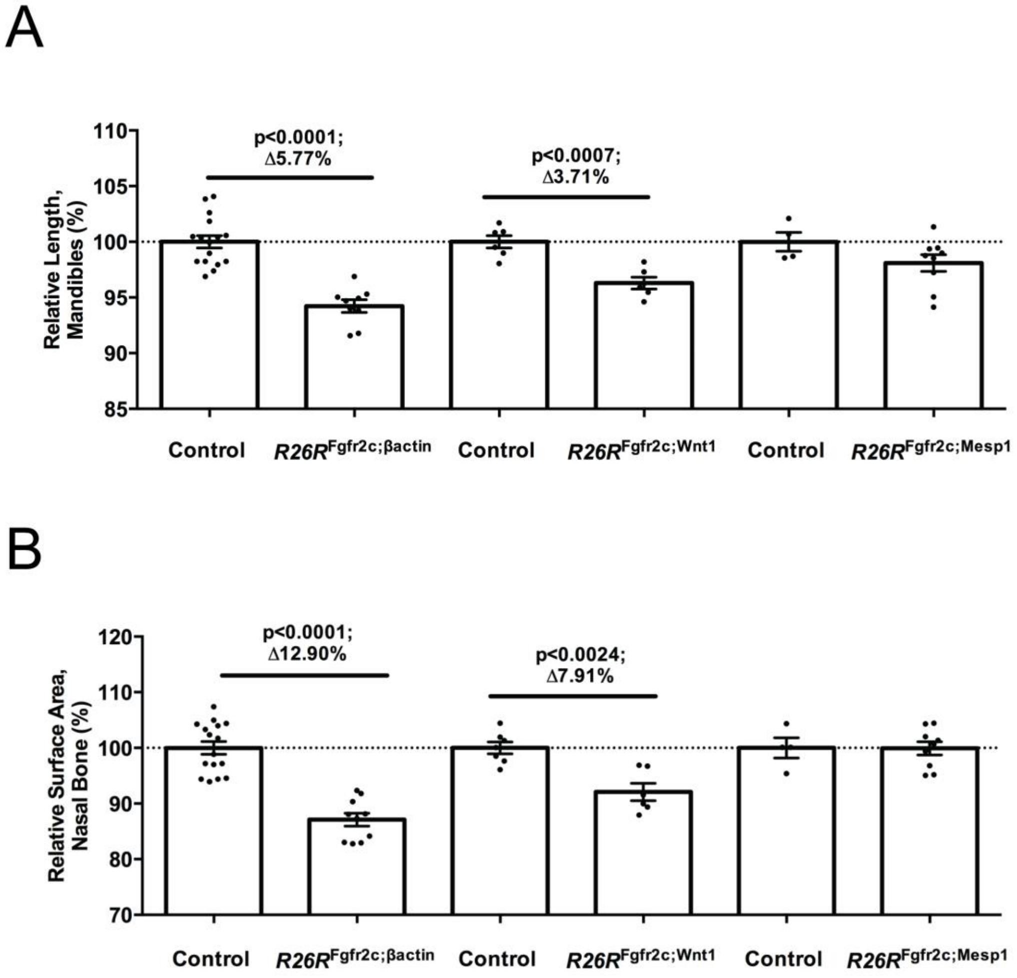

Figure S4. Quantitative analysis of neural crest derivatives in the craniofacial skeleton shows a decrease in size of mandible (**A**) and nasal bones (**B**) in *R26R*$^{Fgfr2c;βact}$ and *R26R*$^{Fgfr2c;Wnt1}$ E18.5 embryos. Statistics: Student's t-test with Welch's correction.

Disease Models & Mechanisms • Supplementary information
